# Following specific podocyte injury captopril protects against progressive long term renal damage

**DOI:** 10.12688/f1000research.4030.1

**Published:** 2015-06-29

**Authors:** Yu S Zhou, Ihmoda A Ihmoda, Richard G Phelps, Christopher OS Bellamy, A Neil Turner

**Affiliations:** 1 Centre for Inflammation Research, Renal Medicine, University of Edinburgh and Royal Infirmary, Edinburgh, EH16 4SB, UK

**Keywords:** ACEi, angiotensin, podocyte, proteinuria

## Abstract

Background: Angiotensin converting enzyme inhibitors (ACEi) reduce proteinuria and preserve kidney function in proteinuric renal diseases. Their nephroprotective effect exceeds that attributable to lowering of blood pressure alone. This study examines the potential of ACEi to protect from progression of injury after a highly specific injury to podocytes in a mouse model.

Methods: We created transgenic (Podo-DTR) mice in which graded specific podocyte injury could be induced by a single injection of diphtheria toxin. Transgenic and wild-type mice were given the ACEi captopril in drinking water, or water alone, commencing 24h after toxin injection. Kidneys were examined histologically at 8 weeks and injury assessed by observers blinded to experimental group.

Results: After toxin injection, Podo-DTR mice developed acute proteinuria, and at higher doses transient renal impairment, which subsided within 3 weeks to be followed by a slow glomerular scarring process. Captopril treatment in Podo-DTR line 57 after toxin injection at 5ng/g body weight reduced proteinuria and ameliorated glomerular scarring, matrix accumulation and glomerulosclerosis almost to baseline (toxin: 17%; toxin + ACEi 10%, p<0.04; control 7% glomerular scarring). Podocyte counts were reduced after toxin treatment and showed no recovery irrespective of captopril treatment (7.1 and 7.3 podocytes per glomerular cross section in water and captopril-treated animals compared with 8.2 of wild-type controls, p<0.05).

Conclusions: Observations in Podo-DTR mice support the hypothesis that continuing podocyte dysfunction is a key abnormality in proteinuric disease. Our model is ideal for studying strategies to protect the kidney from progressive injury following podocyte depletion. Demonstrable protective effects from captopril occur, despite indiscernible preservation or restoration of podocyte counts, at least after this degree of relatively mild injury.

## Introduction

Podocytes are terminally differentiated, highly specialised epithelial cells which cover the outer surface of the glomerular basement membrane and form the final barrier to protein loss during glomerular filtration. Podocyte dysfunction and subsequent loss plays a major role in the initiation and progression of glomerular diseases ^1, 2^. Podocyte injury is characterised by leakage of protein into urine, which can occur even without morphological changes detectable by light microscopy. The close association between damaged podocytes and proteinuria is supported by the observations that numerous congenital causes of substantial proteinuria are due to mutations influencing podocyte-specific molecules such as molecules involved in the cytoskeleton ^3^ and slit diaphragm (podocin, nephrin and CD2AP ^4– 7^).

Kriz and colleagues ^1^ proposed that progressive podocyte damage might lead to chronic renal failure in a number of renal diseases, and that progression might arise because “podocyte damage damages podocytes” (directly or through loss of inter-cell support), leading to a vicious cycle that drives progressive glomerular injury and scarring ^8, 9^. If so, interventions that reduce the disruption by rescuing susceptible podocytes next to injured ones are potential therapies to restore podocyte phenotype and therefore ameliorate renal damage and/or protect the kidney from progressive damage.

There is strong evidence that proteinuria reduction with angiotensin converting enzyme inhibitors (ACEi) and angiotensin receptor blockers (ARB) can arrest deterioration in renal function in proteinuric kidney diseases of any aetiology, both in animal models ^10– 13^ and in man ^14, 15^. This protection was first attributed to haemodynamic effects. Recent evidence suggests additional mechanisms ^16, 17^.

We developed a model of targeted podocyte injury by constructing a transgenic mouse (Podo-DTR) in which the diphtheria toxin receptor is expressed on podocytes. Murine cells are naturally 1000-fold less susceptible to diphtheria toxin than human cells ^18^. Transgenic mice with podocytes fully susceptible to diphtheria toxin were made by expressing the human diphtheria toxin receptor (hDTR, also known as human heparin binding epidermal growth factor receptor, HB-EGFR) under the control of a fragment of the nephrin promoter that was previously shown to be expressed solely in podocytes when coupled to β-galactosidase ^19^. This technique was first applied to hepatocytes ^20^ but has subsequently been successfully applied to a number of other cell types ^8, 21^.

Unlike models of podocyte injury that involve use of toxins of uncertain specificity, such as adriamycin in mice ^22, 23^, puromycin in rats ^24^, and possibly pamidronate toxicity and HIV infection in humans ^25^, our Podo-DTR model permits a graded, specific podocyte injury that can be delivered by a single injection of diphtheria toxin. Here we describe application of Podo-DTR to investigate the nephroprotective potential of ACEi in podocyte injury.

## Methods

### Generation of Podo-DTR mice

The HB-EGF receptor was expressed as a transgene under the control of the murine nephrin promoter. A murine 1.25kb nephrin gene fragment ^19^ was generated from murine genomic DNA. Oligonucleotide PCR primers (MWG Oligo synthesis) for the mouse nephrin gene with added NotI and BamHI restriction sites (primer sequences: 5'-ATGGCCCAGGGATTCAGGTGC-3' and 5'-GCTTGGACCCAGTGTGAACTC-3') were used to clone the gene fragment. The thermal cycling protocol of the PCR machine (Thermal Cycler Apollo ATC201) comprised an initial denaturation step at 75ºC for 10 minutes followed by 30 cycles of 95ºC for 1minute (denaturation), 60ºC for 1 minute (annealing) and 72ºC for 1.5 minute (elongation). The final cycle consisted of a re-annealing at 72ºC for 10 minutes.

This nephrin fragment was used to replace the albumin promoter in the pMS7 plasmid, which was kindly donated by Dr. Saito ^20^. The resulting plasmid consisted of the murine nephrin promoter-fragment and the human HB-EGF cDNA ^19^. Expression driven by this nephrin promoter fragment was shown by Moeller *et al.* (2002) to achieve podocyte specific expression in kidneys without detectable expression outside the kidney by chemiluminescence assay.

**Figure 1.**
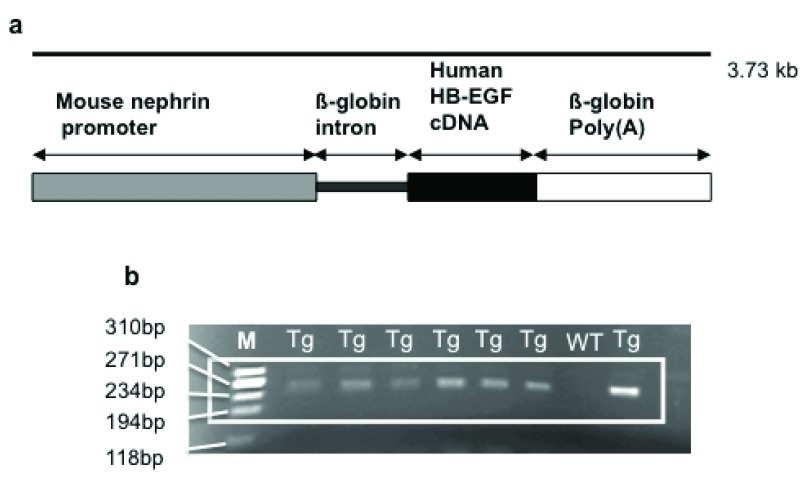
( **a**) Schematic representation of the transgene. Human diphtheria toxin receptor (DTR), or human heparin binding-epidermal growth factor (HB-EGF) receptor, is expressed as a transgene under the control of the murine nephrin promoter. ( **b**) Agarose gel showing positive bands for the PCR products of a 243bp fragment from podocyte nephrin promoter and rabbit-ß-globin intron of transgenic Podo-DTR mice. M, marker Hae ladder; *Tg*, trasgenic; *WT*, wild type.

Transgenic mice were generated by male pronuclear microinjection of murine fertilized ova derived from B6CBAF1 mice with the linearised pIN plasmid ( Figure 1). These mice originated from Harlan UK, and were offspring of a cross between the C57BL/6JolaHsd inbred female and the CBA/CaOlaHsd inbred male.

Transgenic offspring were identified by PCR analysis of ear notch DNA using the following primers: 5'-GGA AGA GAG AAG GGC GAG TT-3' and 5'-GGG TCC ATG GTG ATA CAA GG-3' for a 243bp nephrin gene/intron product; and 5'-GGT GGT GCT GAA GCT CTT TC-3' and 5'-GCT TGT GGC TTG GAG GAT AA-3' for a human HB-EGF gene product. Thermal Cycler Apollo ATC201 PCR machine was used with the same thermal cycling protocol as described above except for the annealing temperature (set to 50ºC for 1 minute per cycle).

All animal studies conformed to local ethical guidelines and the Home Office (UK) Animals Scientific Procedures Act (1986) and were approved by the University of Edinburgh Ethical Review Committee (Ethical Review Number PL23-07). Mice were housed in laboratory cages (n=1–6) in a room with 12:12 hour light-dark cycle and allowed free access to standard dry pellet diet and water (unless otherwise specified) in accordance with the institutional guidelines.

### Characterisation of the model

Podo-DTR mice and wild-type littermates as controls were injected with a single dose of diphtheria toxin (DT) (Product no. 150, Lot 15023A1; List Biological Laboratories Inc., California, USA) ranging from 0.1–166ng/g body weight (bw). At various time points, urine and blood samples were collected for albuminuria and creatinine, and urea measurement respectively. Animals were sacrificed (aged 7 to 11 months) by intraperitoneal injection of terminal anaesthesia (1mg/ml medetomidine (Dormitor) (Ref. VD DOM02, made by Orion Pharma, supplied by Henry Schein Medical) and 100mg/ml ketamine (Vetalar) (Ref. PD VET10, made by Pfizer, supplied by Henry Schein Medical) at approximately 0.1ml/10g of body weight and their kidneys analysed histologically.

### ACEi protection experiment

Groups of 16 transgenic ( *Tg*) mice and eight wild-type ( *WT*) littermates were given captopril (200mg/L) (Product no. C4042, Sigma Aldrich, UK) in their drinking water or placebo (water alone) 24h after a single intraperitoneal (i.p.) injection of diphtheria toxin at 5ng/g bw (Product no. 150, Lot 15023A1; List Biological Laboratories Inc., California, USA). The size of the experimental group was based on a power calculation in order to have 80% power to detect a histological score difference of 1 on a 5 point scale with p≤0.05, assuming SD for score is 1.

Animals of both sexes (1:3 female to male ratio) aged 3 to 12 months (mean: 7.4, median: 8 months) were allocated to three groups:

- *Tg* DT+H _2_O received water alone;
- *Tg* DT+ACEi were treated with captopril;
- *WT* DT+ACEi were wild-type littermate controls treated with captopril.

Urine samples were collected over 24h in metabolic cages at days 0, 14, 42, 49 and 56 and analysed for albumin:creatinine ratio (ACR). Systolic blood pressures were measured by tail cuff plethysmography on trained conscious animals during week 7 after toxin injection, and the mean of 3–4 measurements was recorded for each animal. Untreated Podo-DTR mice (n=5) (transgenic not given diphtheria toxin or captopril) were also included to assess baseline blood pressure.

Terminal blood samples were collected at week 8 from intraperitoneally anaesthetised animals (injected with medetomidine and ketamine).

Kidneys from each animal were bisected sagitally and fixed as required by overnight incubation at 4ºC in fixative (10% neutral formalin (VWR Brand P/L-Chemicals), Methyl-Carnoy fixative (60% absolute methanol, 30% chloroform, 10% glacial acetic acid (Fisher Scientific UK Ltd), or Karnovsky’s glutaraldehyde (700mOsm) (Ref. G5882-100ml, Sigma Aldrich) or snap frozen in liquid nitrogen. For light microscopy, formalin-fixed samples were embedded in paraffin-wax and 2µm sections cut and stained with haematoxylin and eosin (H&E) or periodic acid-Schiff (PAS) (Fisher Scientific UK Ltd).

### Urine and blood analysis

Urine and serum creatinine concentrations were measured using the creatinase reaction, with the exception of the first cohort of the model evaluation studies where Jaffe reaction was used (for the Podo-DTR line 47 given 1ng/g DT). Serum urea was measured using the urease reaction (Alpha Laboratory Ltd, Poole, UK). An immunoturbidimetric assay was developed to measure urinary mouse albumin concentration using a commercial diagnostic Microalbumin Kit (Olympus Diagnostic Ltd, Watford, UK) standardised against purified mouse albumin (Sigma Chemical Co. Poole, UK). All the assays were adapted for use on a Cobas Fara centrifugal analyser (Roche diagnostics Ltd, Welwyn Garden City, UK) according to manufacturer’s instructions.

### Glomerulosclerosis score

Sclerosis was defined as collapse and/or obliteration of glomerular capillary tuft accompanied by presence of hyaline material and/or an increase in matrix ^12^. Glomerulosclerosis was graded on 2µm thick PAS-stained sections, adopting the semi-quantitative scoring system proposed by El Nahas *et al.* ^26^. The severity of glomerulosclerosis was expressed on a scale of 0 to 2. The scoring system used was as follows: 0: normal glomerulus or no lesion; 1: <50% sclerosis; 2: 50–100% sclerosis of glomerular tuft area. Using light microscopy at a magnification of x40 (Olympus CX40), 100 glomeruli per animal were scored for glomerulosclerosis by an observer blinded to experimental group.

### Immunohistochemistry

Paraffin-embedded, formalin-fixed kidneys were cut at 3µm thickness. After deparaffinising and hydrating, the sections were treated with the antigen retrieval solution Borg Decloaker RTU (Ref. BD1000MM Biocare Medical) for 2 minutes after reaching pressure according to manufacturer’s instructions. The sections were incubated with rabbit polyclonal anti-Wilm’s tumour 1 (WT1) sc-192 IgG antibody (1:50; Santa Cruz Biotechnology, Inc) for 1h at room temperature. Immunoperoxidase staining was performed according to the Vectastain ABC kit (Vector Laboratories). Diaminobenzidine (DAB) (Vector Laboratories, Inc Burlingame, CA) was used as the immunoperoxidase detection system.

### Podocyte quantification and glomerular area measurement

WT-1 is a marker that specifically stains podocyte nuclei. Podocytes were counted in 50 consecutive glomerular cross-sections per animal viewed at x40 magnification (Olympus CX40), and the mean podocyte per glomerulus count calculated for each animal, n=16 (transgenic), n=8 (wild-type).

To investigate for any change in glomerular size following treatment (with diphtheria toxin and/or ACEi treatment), 50 consecutive glomerular cross section (GCS) per animal were measured using ImageJ 1.4r software on pictures taken at x10 magnification using QCapturePro (QImaging Micropublisher 3.3 RTV, Zeiss Axioskop, Germany).

### Statistical analysis

Results are expressed as mean ± standard error of the mean (SEM) or median where specifically stated. Statistical differences between groups were tested by the Student t-test or Wilcoxon Rank Sum as appropriate (Graphpad Prism 4 version 1.0 or R www.r-project.org version 2.1). A p-value of <0.05 was considered to be significant.

## Results

### Derivation

Three transgenic lines with positive PCR results for the transgene ( Figure 1b) were established (Podo-DTR 47, 57 and 21). Earlier studies in total of 10, 17 and 24 mice of Podo-DTR line 47, 57 and 21 respectively (male and female) aged 6.5 to 9 months showed that two of the lines (47 and 57) were susceptible to diphtheria toxin. All three lines were entirely healthy and had normal glomerular morphology. Non-transgenic animals and also animals from line 21 had normal glomerular morphology regardless of the dose of toxin received. Mice from lines 47 and 57 developed renal injury in response to low doses of toxin but no pathology outside the kidneys was detected.

### Podo-DTR mice develop podocyte injury after diphtheria toxin injection

In dose-ranging studies, animals were injected intraperitoneally with diphtheria toxin at 0.1–166ng/g bw. Mice from line 47 were most susceptible and developed fatal acute renal injury in response to doses of 2.5ng/g or greater (n=3–6/group, aged 2–10 months), and a dose-dependent, slowly progressive (over 6 months) glomerulosclerosis in response to lower doses (n=3–6/group, aged 2–6 months). Mice from line 57 developed only proteinuria even at doses as high as 20ng/g bw (n=6/group, aged 2–10 months). Mice from line 21 were unaffected by administration of toxin despite carrying the transgene (n=3–6/group at 1ng and 50ng/g bw, aged 6–9 months).

### Albuminuria and serum urea peak at week 2

**Figure 2.**
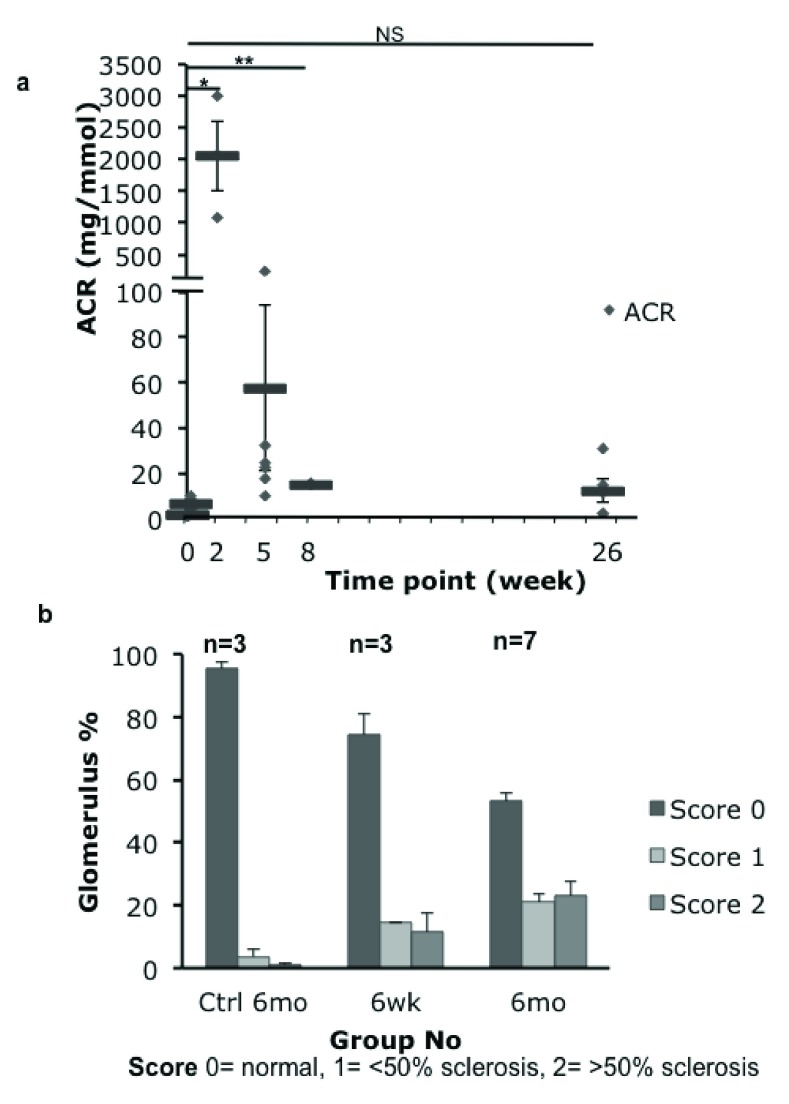
( **a**) Albumin:creatinine ratio (ACR) measured in urine of Podo-DTR line 47 at intervals after injection with 1ng/g bw. Acute proteinuria was highest at week 2 (mean value 2047.9mg/mmol) falling to much lower levels by 5 weeks (57.6mg/mmol) and remaining low at 26 weeks (12.4mg/mmol). No substantial change was seen in control groups (0.9–6.3mg/mmol) (data not visualized). ( **b**) Glomerulosclerosis score of Podo-DTR line 47 mice at 6 and 26 weeks post DT injection at 1ng/g bw. 100 glomeruli were scored per animal (Uncropped gel image in Supplementary Figure 1).

The time course of sub-lethal toxin treatment in line 47 was: development of proteinuria (6mg/mmol at 48h vs 2mg/mmol baseline, p=0.031) within days which increased in severity over 2 weeks (2048mg/mmol) then declined (57.6mg/mmol at 5 weeks) but never to baseline (12.4mg/mmol at 26 weeks (n=3–6) ( Figure 2a). No significant albuminuria was seen in transgenic animals not given toxin or wild-type animals treated with diphtheria toxin (0.9–6.3mg/mmol) ( Supplementary Table 1).

Serum urea showed a similar acute profile to ACR ( Supplementary Table 2). Although the number of experimental animals was small (n=3–7), it appeared that there was an acute rise in blood urea at day 14 (10.9mmol/L vs 5.4mmol/L at d0), followed by temporary recovery at week 5 (5.1mmol/L) and then by slow deterioration (7.9, 8.1, 9.2mmol/L at week 6, 8 and 26 respectively).

### Morphological glomerular damage is progressive

**Figure 3.**
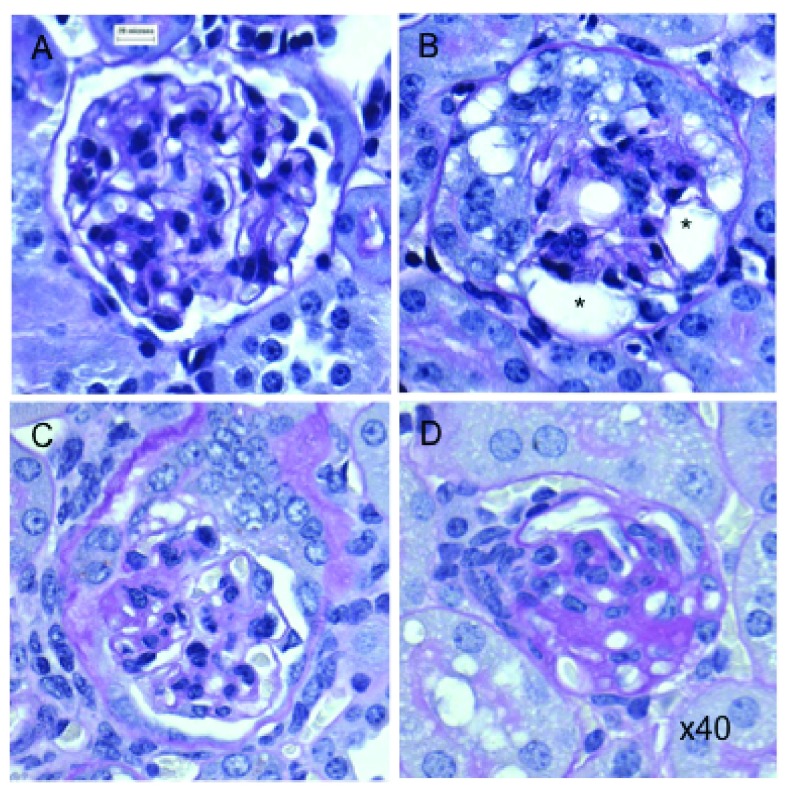
Glomerular histology of DT treated and untreated mice. ( **A**) Normal glomerulus in untreated transgenic mouse. ( **B**) Damaged glomerulus with abnormal morphology and vacuolated cytoplasm (*) from treated Podo-DTR line 47 (25ng/g bw DT) at D7. ( **C**– **D**) Sclerosed glomeruli from treated Podo-DTR line 57 (1ng/g bw DT) at D28 with ( **C**) partially damaged glomerulus with normal morphology at 4 o’clock, with tuft/capsular adhesion in the area of segmental scar at 9 o’clock, and ( **D**) almost completely sclerosed glomerulus. PAS staining, 40x magnification. * p=0.002; ** p<0.0001; DT, diphtheria toxin; bw, body weight; D, day; NS, non significant; PAS, Periodic Acid Schiff.

Toxin treated transgenic (Podo-DTR) mice showed a progressive reduction in the number of normal glomeruli with time (74% at 6 weeks, 53% at 26 weeks, Figure 2b). At 1 month, early focal segmental glomerulosclerosis (FSGS) and chronic renal damage was observed ( Figure 3C–D). Importantly, additional glomeruli became morphologically abnormal even after 6 weeks post-toxin-treatment. In contrast, almost all of sampled glomeruli in control group animals (wild-type toxin-treated and transgenic untreated animals) were morphologically normal (96%) ( Figure 2b).

### Podocyte number falls and remains low

**Figure 4.**
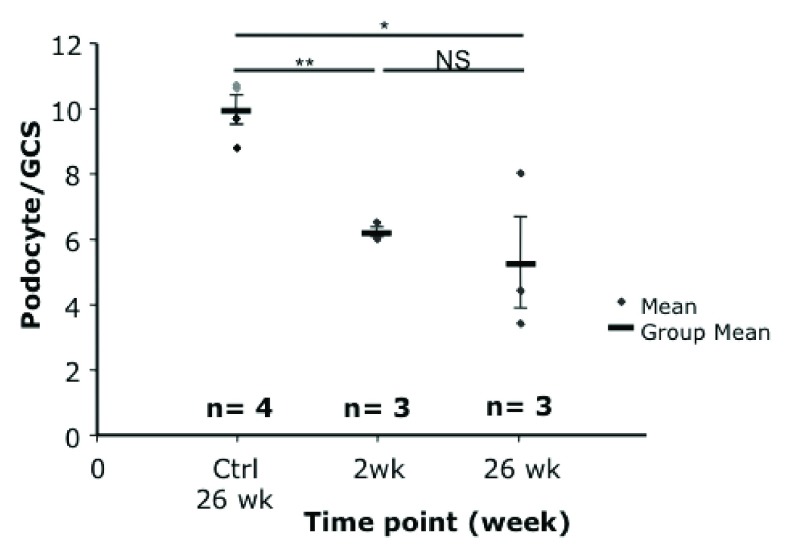
Podocyte quantification of Podo-DTR line 47 mice at 2 and 26 weeks after diphtheria toxin (DT) injection at 1ng/g bw. Podocyte numbers were significantly reduced at 2 and 26 weeks after toxin injection compared to the controls (6.2 and 5.3 versus 10.0 podocyte/GCS respectively, p<0.02). bw, body weight; GCS, glomerular cross section; * p=0.015; ** p=0.001; ♦, wild-type control mice injected with DT; ♦, transgenic mice injected with saline.

The number of podocytes per glomerular cross section (GCS) was reduced in toxin-treated susceptible animals compared to the controls in Podo-DTR line 47 (6.2 at 2 weeks, 5.3 at 26 weeks vs 10.0 podocyte per GCS in controls, p<0.02) ( Figure 4). The reduction in podocyte count was dose-dependent in line 57 animals. At higher toxin dose (20ng/g bw DT) at 14 days, the mean podocyte number dropped to 6.1 podocyte/GCS compared with 7.2 podocyte/GCS in mice treated with lower dose at 5ng/g bw toxin at week 8 versus 8.5 podocyte/GCS of *Tg* saline treated controls (p<0.04). ( Supplementary Table 3).

## Drug intervention study

### ACEi-treatment lowered blood pressure and proteinuria

**Figure 5.**
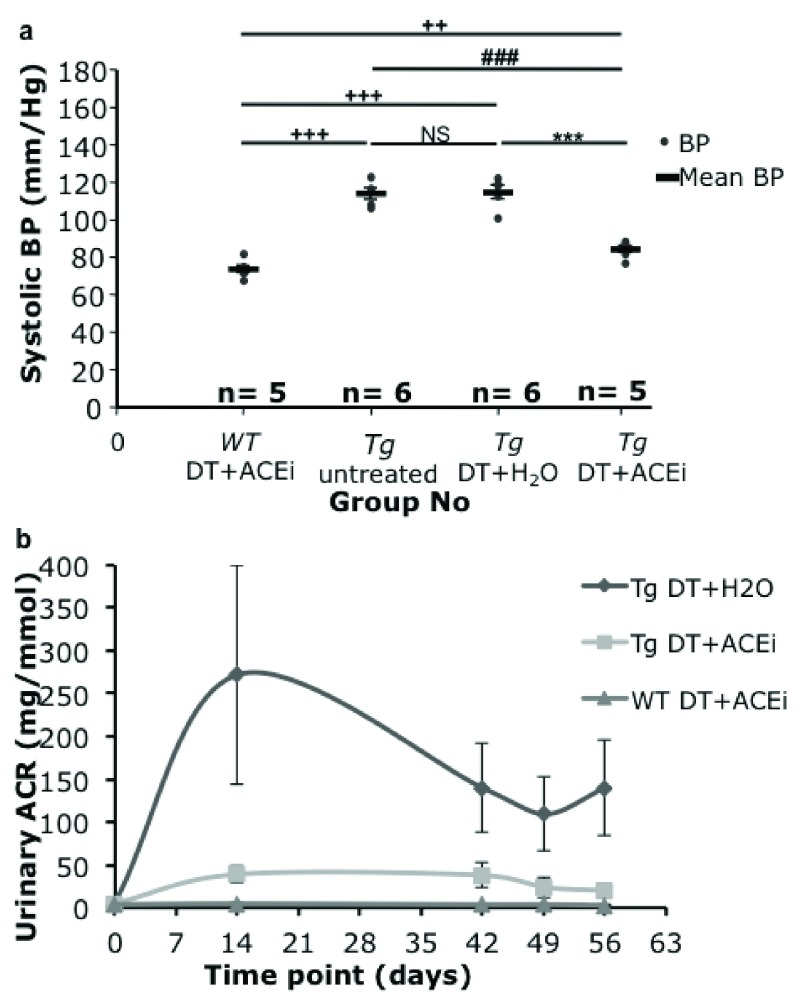
( **a**) Tail cuff blood pressure (BP) of Podo-DTR line 57 mice. At 7 weeks post DT injection (1ng/g bw), the BP of ACEi treated mice, whether *Tg* or *WT* (84 and 73mmHg respectively) were significantly lower (p<0.001) than the untreated groups (114mmHg). ( **b**) Urine albumin:creatinine ratio (ACR) of Podo-DTR line 57 mice. At day 0, prior to DT injection, mice from all 3 groups had baseline level of urine ACR (range: 0.0–6.8mg/mmol). The DT+H _2_O treated group peaked at week 2 (271.5±128mg/mmol), the DT+ACEi treated group had the urinary ACR level blunted substantially (39.1±9mg/mmol). The long-term albuminuria was lowered in both DT+ACEi treated and DT+ H _2_O groups. The *WT* controls had baseline level of urine ACR throughout the experiment (mean range value: 3.0–4.6mg/mmol). DT, diphtheria toxin; ACEi, angiotensin converting enzyme inhibitor; *Tg*, transgenic; *WT*, wild type; bw, body weight; vs, versus; *, p<0.0001 vs *Tg* DT+H _2_O; #, p≤0.0001 vs *Tg* DT+ACEi; +, p=0.002 vs *WT* DT+ACEi.

ACEi-treatment lowered systolic blood pressure in toxin-treated line 57 and wild-type mice from a mean of 114 to 84±1.7 and from 114 to 73±1.9 mmHg respectively ( Figure 5a).

ACEi-treatment also substantially reduced proteinuria in toxin-treated line 57 mice, although not to the levels observed prior to toxin treatment, or in wild type mice (range: 0.0–6.8mg/mmol) ( Figure 5b). The peak level of proteinuria (at week 2) was reduced from 272±128mg/mmol in ACEi-treated mice compared to 39.1±9mg/mmol in mice treated with the diphtheria toxin only, and was substantial at all measurement times ( Figure 5b).

### ACEi-treatment reduced histological damage

**Figure 6.**
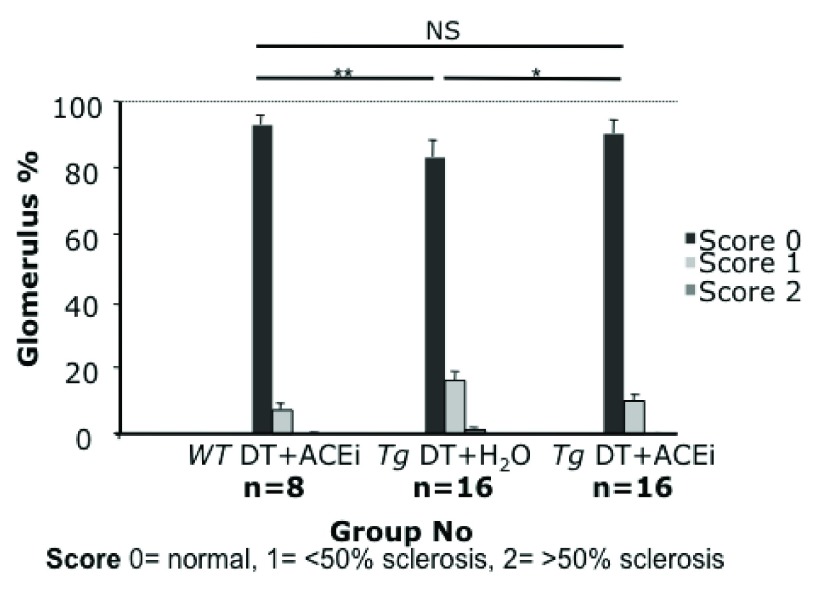
Glomerulosclerosis score of Podo-DTR line 57 mice at 8 weeks post DT injection at 5ng/g bw +/- captopril (200mg/L). Glomerular scarring was reduced almost to baseline level by ACEi captopril ( *Tg* DT+H _2_O: 17%, *Tg* DT+ACEi: 10%, p<0.04; wild-type control: 7%).

**Figure 7.**
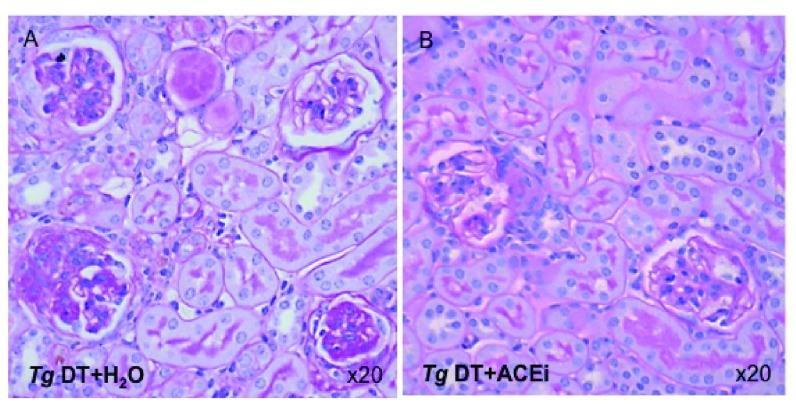
Kidney histology of ACEi treated and untreated Podo-DTR line 57 mice. ( **A**) Cluster of 4 glomeruli with varying degree of scar from a *Tg* DT+ H _2_O treated mouse injected with 5ng/g bw DT; ( **B**) Improved glomerular histology of a *Tg* DT+ACEi treated mouse injected with 5ng/g bw DT. PAS staining, 20x magnification. DT, diphtheria toxin, ACEi: angiotensin converting enzyme inhibitor, bw: body weight; *Tg*, transgenic; *, p<0.02; **, p≤0.001; NS, non significant (p=0.33); PAS, Periodic Acid Schiff.

The proportion of glomeruli showing scarring and matrix accumulation in toxin-treated line 57 mice was substantially reduced in ACEi-treated mice (10% vs 17%, 10%, p<0.04), almost to the levels observed in wild-type control mice (7%) ( Figure 6 & Figure 7).

### ACEi-treatment did not protect from podocyte loss

**Figure 8.**
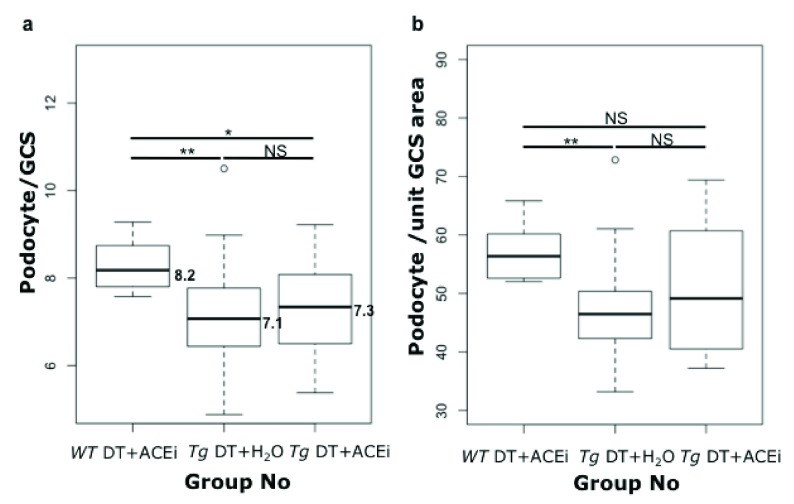
Box plots of the podocyte count. ( **a**) or podocyte count per unit glomerular cross section area ( **b**) for Podo-DTR mice treated with toxin with or without ACEi compared with wild-type (toxin + ACEi treated) mice at week 8. Significance was assessed using the 10% Wilcoxon Rank Sum test. *Tg*, transgenic; *WT*, wild-type; ACEi, angiotensin converting enzyme inhibitor; GCS, glomerular cross section; DT, diphtheria toxin; NS, non significant; *, p=0.03; **, p≤0.003.

Podocyte counts at week 8 were lower in toxin-treated susceptible mice than wild-type controls (median podocytes per GCS was 7.1 for *Tg* DT+H _2_O, and 8.2 for wild-type mice, p<0.05 by Wilcoxon Rank Sum). However, the counts in ACEi-treated, toxin-treated line 57 mice were not significantly higher (7.3 for *Tg* DT+ACEi) than in toxin-treated line 57 mice, and were significantly lower than in wild type controls (10% Wilcoxon Rank Sum) ( Figure 8a). These results suggest that ACEi-treatment was not advantageous for podocyte preservation in this model ( Figure 8b).

A lack of benefit of ACEi treatment for podocyte preservation continued to be apparent when podocyte counts were expressed as counts per unit in glomerular cross sectional area, in order to compensate for any change in glomerular size. Indeed, mean glomerular size measured in 50 consecutive glomerular cross-sections using ImageJ 1.4r software was similar in the experimental groups irrespective of toxin-treatments at 9676, 10708, and 9693µm ^2^ in *WT* DT+ACEi, *Tg* DT+H _2_O and *Tg* DT+ACEi respectively, p>0.08 ( Supplementary Table 4).

## Discussion and conclusions

Our results demonstrate utilisation of mice engineered to be susceptible to podocyte damage to evaluate the capacity of podocyte-protective drugs such as captopril to modulate self-perpetuating mechanisms of podocyte damage. Other groups have developed toxin receptor-mediated conditional podocyte knockout models ^8, 27^ similar to ours, but the application of such models in drug studies is novel.

Our Podo-DTR mouse model has a number of advantages over the existing animal models. Wiggin *et al.* have employed similar technology ^8, 27^ but in rats rather than mice. A mouse model offers greater potential for further analysis because of the rich availability of mouse-specific reagents and large number of existing transgenic mouse lines. Moreover, breeding turnover is greater with a higher litter number, maintenance costs are lower and smaller volumes of toxins or drugs are required for studies.

Unlike the human (h) CD25 mouse model generated by Ichikawa’s group in Japan ^8, 27^, where only relatively short-term timepoints (up to four weeks) have been presented, even after low dose of LMB2 immunotoxin treatment at 0.625ng/g bw, our Podo-DTR mice survived up to six months after 1ng/g bw DT injection in line 47. This allowed analysis of long-term timepoints for histological changes and sclerosis development.

ACEi and ARB were originally thought to effect renal protection via haemodynamic effects and reduced glomerular filtration pressure. Although blood pressure reduction by any means has been shown to protect renal excretory function in proteinuric diseases ^28^, this accounts for only a part of the activity of ACEi and ARB agents. Proposed additional mechanisms of renal protection include modulation of the toxicity of filtered protein by influences upon non-glomerular cells ^29^: it is thought that filtered protein may be toxic to renal tubular cells contributing to interstitial fibrosis ^30^.

An alternative hypothesis is that ACEi and ARB exert a beneficial influence on the rate of continuing podocyte damage/loss thought to be central to the progression of proteinuric renal diseases and development of chronic renal failure ^8, 9, 31^. There is evidence - from elegant work utilising genetic chimeras in which a podocyte subpopulation expresses the hCD25 toxin receptor and may be selectively injured - that podocyte injury initially focused on a subset of podocytes can spread to other podocytes ^32^. The initial insult caused by the toxin exposure to hCD25-positive podocytes lead to cell death and massive proteinuria within 4 days, followed by a secondary wave of injury to podocytes lacking the toxin receptor at 6 weeks post-toxin administration, with foot process effacement and downregulation of podocyte specific markers such as nephrin, podocin and VEGF in conjunction with an increase of the injury marker desmin. These results parallel the findings of our study where additional glomerular damage and possibly further reduction in podocyte number was observed from up to 26 weeks after acute injury, long after the initial injury induced by diphtheria toxin ( Figure 2b & Figure 4). This suggests that we may also be seeing the phenomenon of propagation of podocyte injury, “podocyte damage damages podocytes” leading to progressive glomerular injury and scarring. This finding may open up the possibility that drugs that protect podocytes may also be generally nephroprotective.

Any broader implication of podocyte health for renal preservation is important to define as many drugs employed in renal disease have been shown to have direct effects on podocytes. As well as ACEi and ARB ^10, 16, 17^, direct effects on podocytes have been reported for: immunosuppressive drugs including corticosteroids ^33, 34^, calcineurin inhibitors such as cyclosporine ^35^ and tacrolimus ^36^, and mizoribine ^37, 38^; and non-immune drugs including the endothelin A receptor antagonist (ETA-RA) ^39, 40^, and peroxisome proliferator-activated receptor gamma (PPARγ) agonists ^41, 42^.

The Podo-DTR mouse model described here has advantages for studying podocyte rescue as it is possible - through selection of the dose of toxin - to produce a consistent non-lethal degree of podocyte-specific injury. Mild degrees of injury were shown to superficially heal over 8 weeks with normalisation of glomerular morphology observable under light microscopy and reduction of proteinuria, but also with continuing reduction in podocyte numbers and low level proteinuria. On this background we were able to demonstrate a benefit of captopril in arresting histopathological progression of glomerular damage despite a reduction in podocyte number. This is in contrast to studies that have shown a protective effect of ACEi upon podocyte number in certain settings ^10, 17^, but in agreement with two other chronic models of renal disease in subtotally nephrectomised rats ^13^ or antibody-induced nephritic mice ^42^.

Comparison of the change in podocyte number between studies is complicated by the range of indirect measurement techniques that have been used. Our study employed quantification of podocytes per glomerular tuft in a 2D image similar to the approach of many other groups ^43– 45^. Another approach is to estimate the total number of podocytes per whole glomerulus by extrapolating a volume from a series of 2D images ^2^, which may have some advantages but is elaborate, very difficult to apply to experiments with large numbers of animals, and assumes glomeruli have a consistent size and spherical shape. The best technique with which to quantify podocytes is still a matter of debate, but whilst this difficulty complicates comparison between studies, it does not prevent conclusions from the comparisons made within the various studies where measurements have been made using a consistent technique.

Our results suggest that protection against podocyte loss is not the only mechanism by which ACEi achieve long-term nephroprotection: it is likely that an influence on podocyte phenotype or function is also important. Our model could be used to identify or screen new compounds to reduce podocyte damage and preserve renal function.

## Supplementary materials

### Supplementary Table 1.

24h urine analysis measuring albumin and creatinine of untreated control Podo-DTR line 47 mice and treated with saline or DT at 1ng/g bw

### Supplementary Table 2.

Serum creatinine and urea concentration of experimental Podo-DTR line 47 mice at various time points

### Supplementary Table 3.

Podocyte quantification of Podo-DTR mice treated with 20ng/g bw DT at week 2 (14d) and 5ng/g bw DT at week 8 (56d). At higher toxin dose (20ng/g bw DT) at 14 days, the mean podocyte number dropped to 6.1 podocyte/GCS compared with 7.2 podocyte/GCS in mice treated with lower dose at 5ng/g bw toxin at week 8 versus 8.5 podocyte/GCS of *Tg* saline treated controls (p<0.04).

### Supplementary Table 4.

Glomerular area and diameter measurements of Podo-DTR line 57 treated with 5ng/g bw DT and ±ACEi captopril (200mg/L). Glomerular area of 50 consecutive GCS was measured using ImageJ 1.4r software. The results showed no significant difference in mean glomerular area between the WT DT+ACEi, *Tg* DT+H _2_O and *Tg* DT+ACEi groups (group mean 9676, 10708, 9693µm ^2^ respectively), p>0.08.

DT, Diphtheria toxin; ACEi, angiotensin converting enzyme inhibitor; GCS, glomerular cross section; WT, wild type; *Tg*, transgenic.

### Supplementary Figure 1.

Raw gel image for Figure 1b.
