# Supplementary figures and images for: Following specific podocyte injury captopril protects against progressive long term renal damage
Source: F1000Res. 2015 Jun 29;4:172. [Version 1] doi: 10.12688/f1000research.4030.1 (PMC4642846; doi:10.12688/f1000research.4030.1)

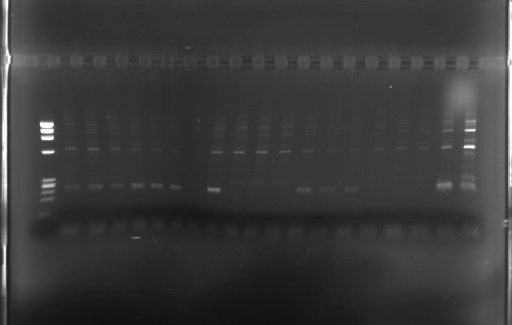

Supplement: Supplementary file 5 [file f1000research-4-4317-s0004.tgz › e2f11cd7-c69c-41c3-8df8-f35912ee927d.tiff]
