# Supplementary material for: Following specific podocyte injury captopril protects against progressive long term renal damage
Source: F1000Res. 2015 Jun 29;4:172. [Version 1] doi: 10.12688/f1000research.4030.1 (PMC4642846; doi:10.12688/f1000research.4030.1)
Supplement: Peer review history

# Peer review: Referee response for version 1

**DOI:** 10.5256/f1000research.4317.r10890

**Published:** 2015-11-02

**Authors:** Gavin I. Welsh

**Affiliations:** 1 Academic and Children’s Renal Unit, School of Clinical Sciences, University of Bristol, Bristol, UK

This is an interesting and well-presented paper that uses transgenic (Podo-DTR) mice to demonstrate (1) that continuing podocyte dysfunction is a key abnormality in proteinuric disease and (2) following specific podocyte injury captopril protects against progressive long-term renal damage. However there are a number of issues with the manuscript.

1. Three transgenic lines with positive PCR results for the transgene were established (Podo-DTR 47, 57 and 21) by the authors two of which (47 and 57) were susceptible to the DT. However instead of comparing the 2 lines with wild type mice, different lines are used for different experiments. The model characterization uses mainly Line 47 but experiments using captopril are done exclusively in Line 57. What happens to mice from Line 47 treated with captopril-does it have the same protective effect to that seen in Line 57?
2. The authors should confirm that the DTR protein is indeed exclusively expressed in the podocyte.
3. The authors do not explain why there is such a wide spread in the ages of the mice (3-12 months) used. Does not ageing introduce unneeded variability into the study that may confound some of the effects seen?
4. There is variability in the doses of DT used in each experiment that makes the results difficult to compare. For example in figure 5 for tail cuff blood pressure and urine albumin:creatine ratio 1ng/g bw was used but for glomerulosclerosis and kidney histology studies animals were treated with 5ng/g bw.  Furthermore kidney histology for Line 57 in figure 3 uses 1ng/g bw but in figure 7 it is 5ng/g bw. Why the lack of consistency?
5. In figure 4 control mice for PodDTR line 47 are reported to have 10 podocytes/GCS whilst those for PodDTR line 47 in figure 8 have 8.2 podocytes/GCS. Why the discrepancy between the two lines?
6. At what age were the samples taken for figure 6 and 7 and what dose of DT was used in figure 8?

I have read this submission. I believe that I have an appropriate level of expertise to confirm that it is of an acceptable scientific standard, however I have significant reservations, as outlined above.

# Peer review: Referee response for version 1

**DOI:** 10.5256/f1000research.4317.r10200

**Published:** 2015-10-26

**Authors:** Johannes Schlondorff

**Affiliations:** 1 Division of Nephrology, Department of Medicine, Beth Israel Deaconess Medical Center, Boston, MA, UK

In their manuscript, the authors adapt the use of podocyte-specific ablation using transgenic diphtheria toxin receptor (DTR) expression to the mouse. The murine model functions similarly to the previously described rat model of Wiggens *et al.* Using the model, the authors examine the role of ACE inhibition (captopril) as a protective therapy. Overall, the conclusions are well supported by the experimental results, though the experiments are neither as detailed nor as compelling as those previously published by others. The two major conclusions of the manuscript, that podocyte depletion can lead to the initiation of a feed-forward podocyte pathology and that ACE inhibition can slow or ameliorate this process, have been well documented by others.

The major concerns about the interpretation of the results stem from the fact that: 1. two different transgenic lines are used without clear side-by-side comparison of both lines for the most relevant experiments; and 2. the animals used in the captopril experiment are mixed sex and of a very large range of ages (3-12 months), likely leading to the large variability in the data, limiting power, and making it difficult to lend much credence to the negative results regarding captopril’s effect on podocyte numbers. Analysis of the data using matching for age and sex, if possible, would be of interest. Overall, the 57 transgenic lines used in the ACEi experiment show only very modest glomerular sclerosis compared to controls (Figure 6), and the control animals in this experiment have surprisingly lower number of podocytes per glomerulus (8.2; figure 8) compared to control animals in an earlier experiment (10.0, figure 4). Some explanation should be provided.

Minor points:

1. Figure 3 legend refers to p values, body weight to which there are no corresponding data presented.
2. Figure 5a: Is there a difference between the “++” and “+++”?  For figure 5b, the lines connecting the time points should be removed. In addition, including individual values and means (as in other figures) or at least including the n’s would be helpful.
3. Number of animals/samples should be consistently provided in the figures (eg figure 8, 5b).

I have read this submission. I believe that I have an appropriate level of expertise to confirm that it is of an acceptable scientific standard, however I have significant reservations, as outlined above.

# Peer review: Referee response for version 1

**DOI:** 10.5256/f1000research.4317.r9900

**Published:** 2015-08-24

**Authors:** Taiji Matsusaka

**Affiliations:** 1 Division of Nephrology, Endocrinology and Metabolism, Department of Internal Medicine, School of Medicine, Tokai University, Isehara, Japan

In this study, the authors established a new mouse model of inducible podocyte injury. They also showed that ACEi treatment effectively attenuated proteinuria and glomerulosclerosis in this model. Similar to previous reported models, this model also reproducibly develops progressive glomerulosclerosis. The data are confirmative, but this model will be an alternative useful model for podocyte injury. Compared to hCD25 transgenic mouse model (NEP25), podocyte injury is relatively mild. The time course of ACR in line 47 (Figure 2a) appears to be similar to that of NEP25 injected with 0.625 ng/g BW of LMB2.

Generally, the study is well done, and the data are properly presented. There are only some minor concerns.

1. It would be better to present HB-EGF mRNA or protein expression data. Is the transgene actually expressed selectively in podocytes?  Is the sensitivity of toxin parallel to transgene expression among three lines?
2. Data among three or more groups should be analyzed by ANOVA or some other methods to reduce type 1 error. Generally, urinary ACR shows logarithmic normal distribution. These data should be properly transformed before analysis. Figure 5b data should be analyzed by mixed effect model or other appropriate methods.
3. In wild-type C57BL/6 mice younger than 10 months of age, glomerulosclerosis is very rare. The wild-type control mice treated with ACEi, 7% of glomeruli showed segmental sclerosis. Is this senile change? If so, more detailed information of mouse age would be presented, or consider deleting aged mice from the experiment.
4. Please indicate what the bottom and top of the box and whisker represent in Figure 8.
5. The authors state that ACEi was not advantageous for podocyte preservation. I do not agree on this statement. It is just a matter of statistical power. The protective effect by ACEi is small relatively to the variance of podocyte number in each group.  

I have read this submission. I believe that I have an appropriate level of expertise to confirm that it is of an acceptable scientific standard.
